# Exploring bacteria-induced growth and morphogenesis in the green macroalga order Ulvales (Chlorophyta)

**DOI:** 10.3389/fpls.2015.00086

**Published:** 2015-03-03

**Authors:** Thomas Wichard

**Affiliations:** Institute for Inorganic and Analytical Chemistry, Jena School for Microbial Communication, Friedrich Schiller University JenaJena, Germany

**Keywords:** axenic cultures, Bacteroidetes, chemical ecology, cross-kingdom interactions, macroalgae, morphogenesis, Roseobacter, seaweed

## Abstract

Green macroalgae, such as Ulvales, lose their typical morphology completely when grown under axenic conditions or in the absence of the appropriate microbiome. As a result, slow growing aberrant phenotypes or even callus-like morphotypes are observed in Ulvales. The cross-kingdom interactions between marine algae and microorganisms are hence not only restricted by the exchange of macronutrients, including vitamins and nutrients, but also by infochemicals such as bacterial morphogenetic compounds. The latter are a fundamental trait mediating the mutualism within the chemosphere where the organisms interact with each other via compounds in their surroundings. Approximately 60 years ago, pilot studies demonstrated that certain bacteria promote growth, whereas other bacteria induce morphogenesis; this is particularly true for the order of Ulvales. However, only slow progress was made towards the underlying mechanism due to the complexity of, for example, algal cultivation techniques, and the lack of standardized experiments in the laboratory. A breakthrough in this research was the discovery of the morphogenetic compound thallusin, which was isolated from an epiphytic bacterium and induces normal germination restoring the foliaceous morphotypes of *Monostroma*. Owing to the low concentration, the purification and structure elucidation of highly biologically active morphogenetic compounds are still challenging. Recently, it was found that only the combination of two specific bacteria from the Rhodobacteraceae and Flavobacteriaceae can completely recover the growth and morphogenesis of axenic *Ulva mutabilis* cultures forming a symbiotic tripartite community by chemical communication. This review combines literature detailing evidences of bacteria-induced morphogenesis in Ulvales. A set of standardized experimental approaches is further proposed for the preparation of axenic algal tissues, bacteria isolation, co-cultivation experiments, and the analysis of the chemosphere.

## Introduction

Morphogenesis in multicellular organisms is a strictly controlled process where cells with identical genetic information are defined by their spatial relation to each other. During morphogenesis, they develop different morphological, biochemical, and functional characteristics driven by inter-, intra-, and extracellular processes (Hurd et al., 2014). The environment of a macroalga is hereby defined by abiotic and biotic parameters and stimuli that dictate the extracellular effects on the growth and development of macroalgae.

This review combines literature detailing evidence of bacteria-induced morphogenesis in Ulvales since 1934, when Føyn stated that the normal growth of *Ulva* and *Cladophora suhriana* was not possible until soil extracts were added (Erdschreiber's culture medium, UTEX, 2014^1^). Since that time, several synthetic media have been developed to study nutrient requirements of macroalgae and to manipulate the additives of growth media allowing normal germination/development until at least the 20-cell stadium of *Ulva lactuca* (Harvey, 1933; Føyn, 1934; Kylin, 1941; Levering, 1946; Provasoli, 1958a,b,c).

**Figure 1:**
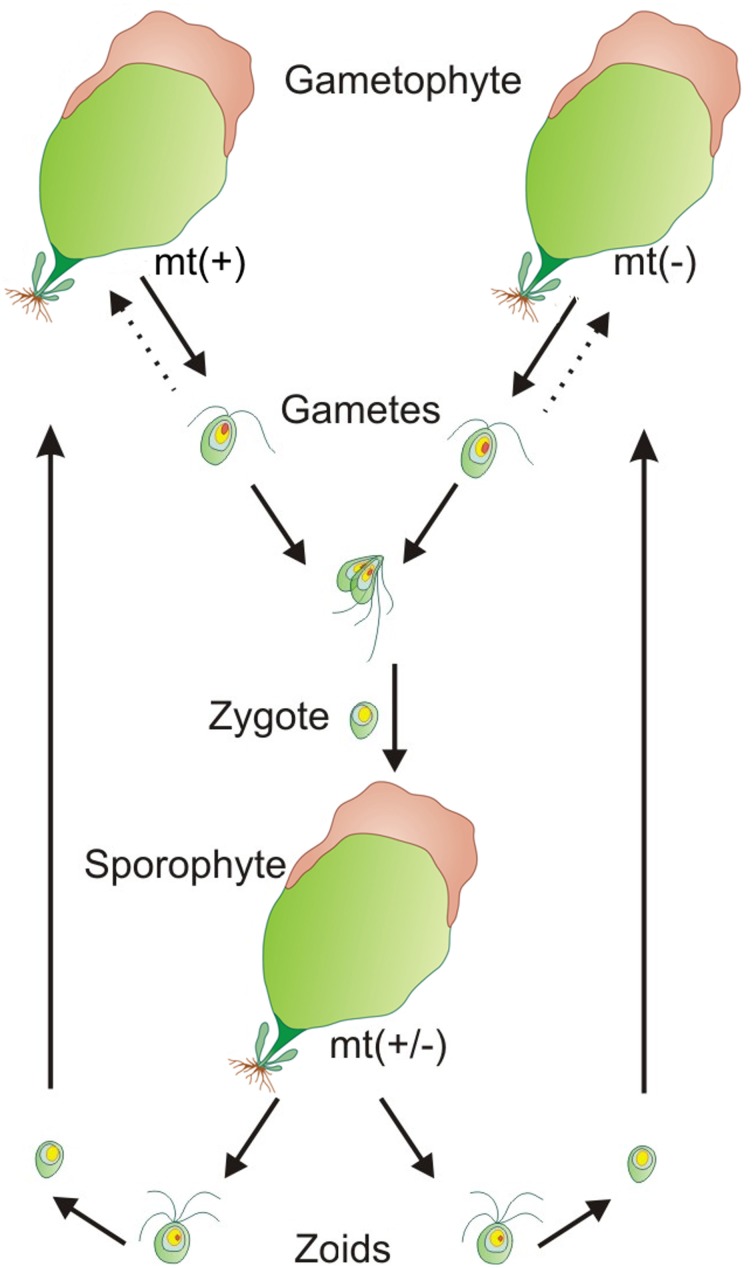
**Isomorphic life cycle of *Ulva***. All *Ulva* species are isomorphic and alternate between gametophytic and sporophytic life stages with similar morphologies. The gametophytes are haploid (n) and the sporophytes are diploid (2n). The mating types (mt) are indicated by (+) and (−). Dashed arrows show the parthenogenetic development of gametophytes derived from unfused gametes.

Understanding of *Ulva*'s life cycle was an further important step on the way to standardized laboratory cultures: All *Ulva* species are isomorphic and alternate between gametophytic and sporophytic life stages with similar morphologies. The gametophytes are haploid and the sporophytes are diploid. The gametophytes produce biflagellated haploid gametes through mitosis, and the sporophytes produce quadriflagellated haploid zoospores (= zoids) through meiosis. Development of both gametophyte and sporophyte follows the same pattern (Løvlie, 1964; Figure 1). Interestingly, haploid gametophytes are derived either from haploid zoids of sporophytes, from unmated biflagellated gametes, or from zoids of parthenosporophytes (Løvlie and Bryhni, 1978; Phillips, 1990). Reproductive activities occur most often near the margins of *Ulva* fronds or at damaged parts of the thalli (Nilsen and Nordby, 1975; Stratmann et al., 1996). The conversion of blade cells into gametangia/sporangia is hereby regulated by two sporulation inhibitors: a high molecular weight cell wall glycoprotein and a low molecular weight factor located in-between *Ulva*'s bilayer, demonstrated for *U. mutabilis* and *U. linza* (Stratmann et al., 1996; Vesty et al., 2015). The fertile portions of the thalli turn slightly brown and can be easily recognized (Figure 1). The gametogenesis/sporogenesis is induced as soon as both sporulation inhibitors drop down under a certain threshold concentration or they are no longer perceived. Upon removal of a third factor, the swarming inhibitor, which has accumulated during the gametogenesis in the medium, biflagellated, positively phototactic gametes are released in the light (Stratmann et al., 1996; Wichard and Oertel, 2010). With the same timing, the differentiation of blade cell into a gametangium might be initiated by a switch of temperature, as shown for certain tropical *Ulva* species (Carl et al., 2014). Reproduction rhythmicity can be also controlled by moon phases in *U. pseudocurvata* (Lüning et al., 2008).

*Ulva* (flat bilayered blades) and *Enteromorpha* (hollow tubes) differ in their general morphology, and hence, they have been maintained as separated genera, although phylogenetic analysis indicated their close relationship. Indeed, there are no valid criteria to distinguish between these two genera even based on morphology: A given clonal haploid swarmer population from a distromatic alga can give rise to all distromatic algae, all tubular algae or a mixture, showing the high plasticity in morphotypes of the filial generation of *U. linza* (Bonneau, 1977; Provasoli and Pintner, 1980). It was most interesting that *Ulva* and *Enteromorpha* species developed from axenic cultures into the same small colonies of uniseriate branching filaments (see Section The Symbiotic Nature of *Ulva* Growth—A Short History). All these evidences were finally confirmed by an elaborate phylogenetic study based on the Ribulose-1,5-bisphosphate carboxylase/oxygenase large subunit (*rbc*L) gene and internal transcribed spacer (ITS) sequences by Hayden et al. (2003), who concluded, “*Ulva and Enteromorpha are not distinct genera*.” It was suggested that this variability inherent within a genome may be expressed epigenetically without any mutation (Bonneau, 1977; Hayden et al., 2003). Such variations within the genome of *Ulva* can happen; maybe through a series of developmental events under the control of, for example, transcription factors that act as master switches, as originally shown in higher plants (e.g., Wu et al., 2008). First epigenetic analyses were conducted in *Ulva* using protoplasts of *U. reticulata* to detect epigenetic variations in germlings developing in different morphotypes (Gupta et al., 2012). In addition, pilot experiments with a novel established transformation system of germ cells will allow the creation of different morphotypes by insertional mutagenesis (W. Oertel, T. Wichard, and A. Weissgerber, personal communication).

**Figure 2:**
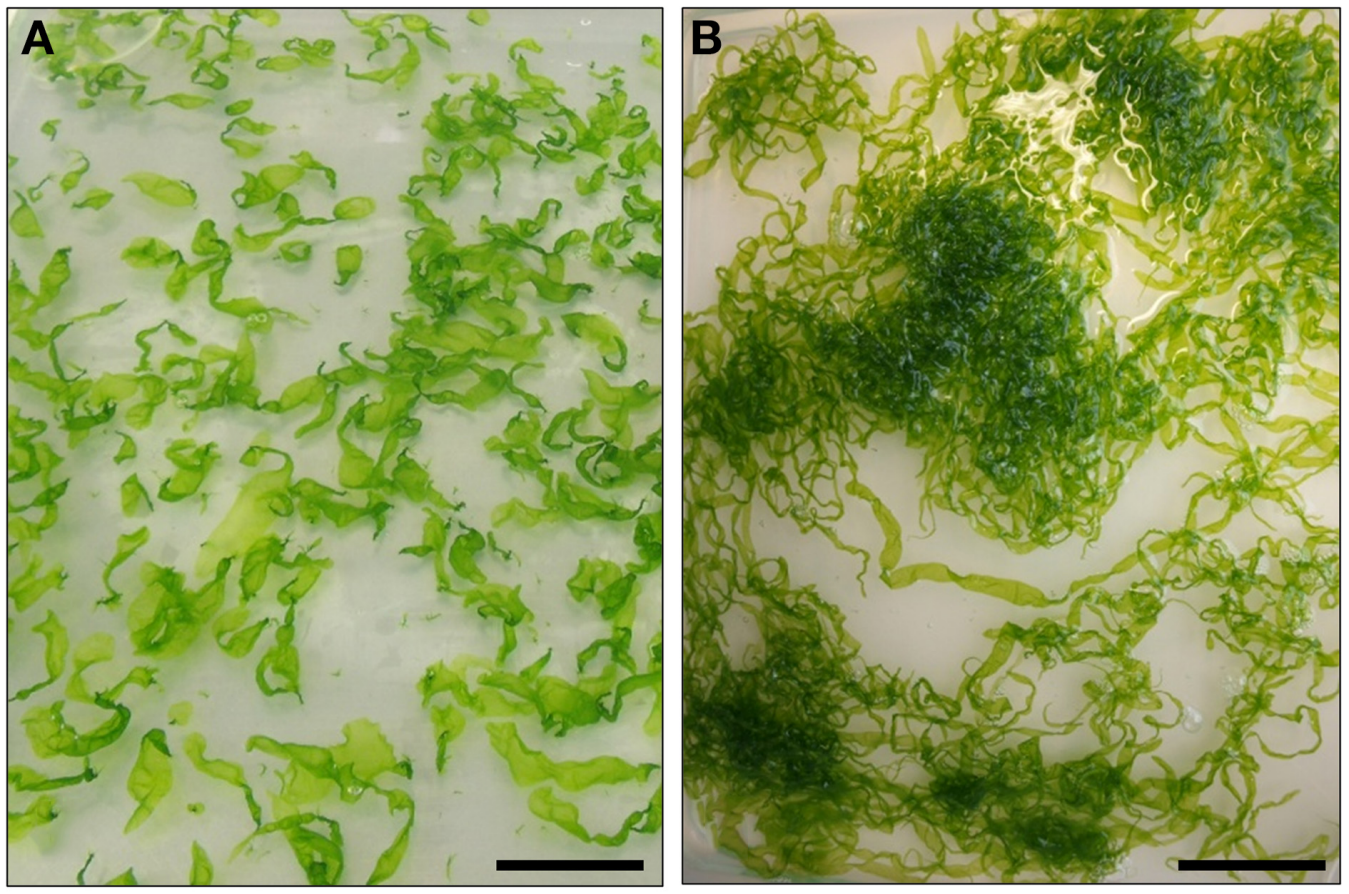
**Typical cultures of the wildtype (A) and the developmental mutant *slender* (B) of *Ulva mutabilis***. The cultures are 5 weeks old. Scale bar = 5 cm.

**Figure 3:**
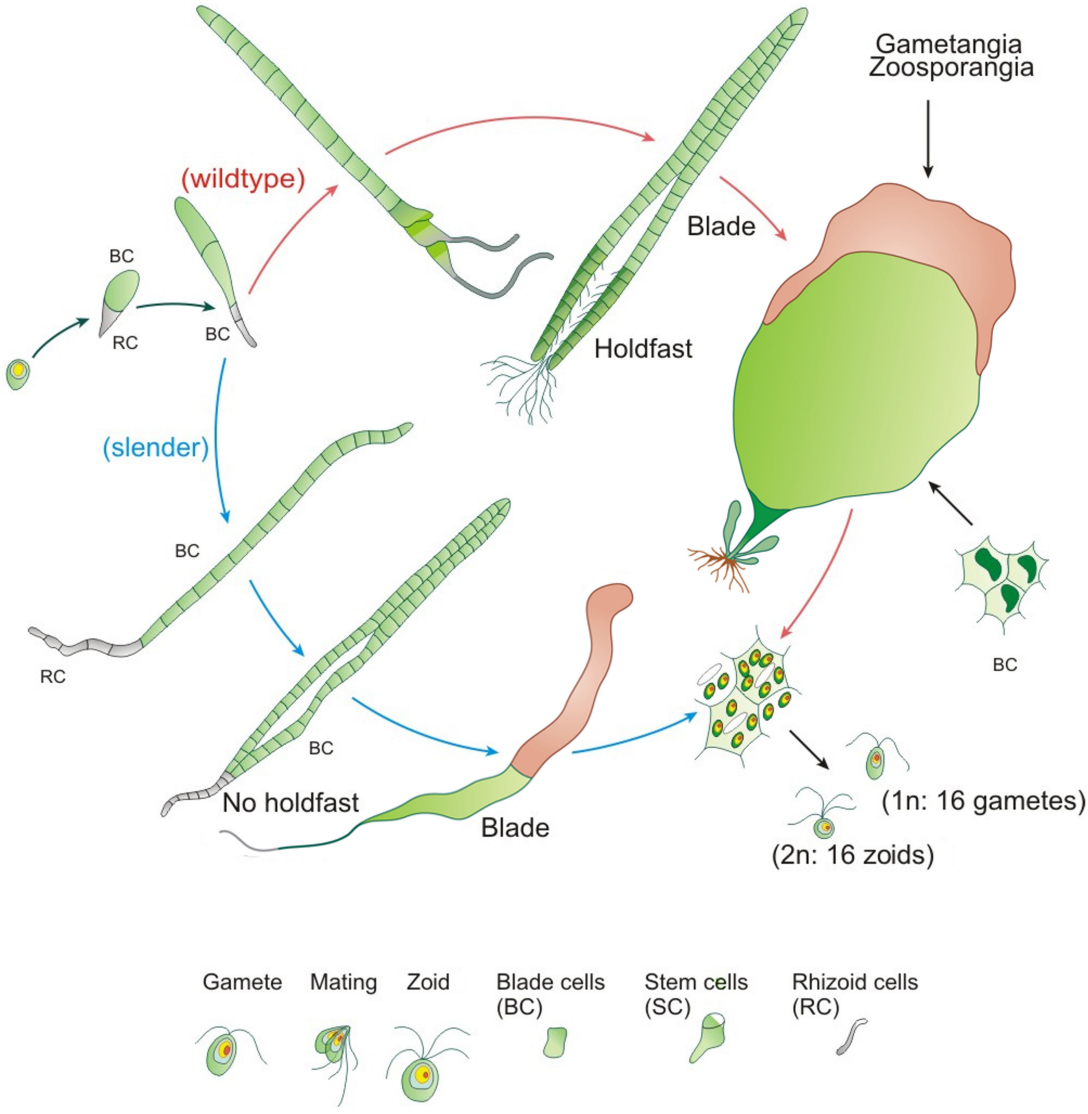
**Development of the cycle of *U. mutabilis***. The wildtype in comparison with the developmental mutant *slender* is shown. Stem cells are colored in dark green (wildtype) and gametangia in brown. Relative sizes are not drawn to scale (adapted, modified and used with permission from Spoerner et al., 2012; Copyright© 2012, Wiley).

*Ulva mutabilis* Føyn (1958) was named “mutabilis,” because of its genetically instability: Certain strains change e.g., spontaneously into different morphotypes, most commonly either a blade-like wildtype or the so-called *slender* morphotype, similar to that observed in the closely related *U. compressa* (Figures 2, 3) (Løvlie, 1968; Tan et al., 1999). Føyn (1958) described a bunch of natural developmental mutants named *slender*, *long*, *branched*, or *bubble* (Fjeld and Børresen, 1975) according to their main morphological characteristics. The fast growing tubular mutant *slender* (monostromatic tubes) is hereby one of the most interesting variations, and it shows only traces of the sea lettuce-like morphotype of the wildtype (Figure 2). Whereas, the wildtype consists of three cell types (blade, stem, and rhoizoid cells) and forms a complete holdfast, *slender* lacks stem cells and develops only primary rhizoids. During the first few days after mating, the zygote increases in volume and becomes pear-shaped with the pointed end directed away from the light (Løvlie, 1964) and surrounded by chemotactically attracted bacteria (Spoerner et al., 2012; see Section *Ulva* Gets What It Needs). On the third day, the first cell division takes place, which differentiates into a primary rhizoid (several elongated cells), which is under the control of specific bacteria, at least in *U. mutabilis*. At the 10-cell stadium, lateral protuberances, from stem cells next to the primary rhizoid, are set off, forming the secondary rhizoid. This differentiation does not occur in the *slender* mutant, as stem cells are missing. In the beginning, all cell division planes are perpendicular to the long axis and all cells are arranged in one row as Løvlie described (1964). When the total number of 20–30 cells is achieved, division parallel to the long axis occurs and the alga becomes basically a tube closed at both ends. The two cell layers of blade cells may partly adhere to each other (Bonneau, 1977, 1978), but in *U. mutabilis*, they are separated, in most cases, by lumen filled with an extractable fluid that contains cell status-regulating metabolites (i.e., sporulation inhibitors; Stratmann et al., 1996). Outgrowths of the inner surface (Figure 3) of the tertiary rhizoid grow backwards inside the alga and emerge at the primary rhizoid, where a group of rhizoids form the holdfast, which is not present in the *slender* mutant. The latter grows in the same fashion as the wildtype until the 10-cell stage. However, the cell division planes, also perpendicular to the long axis, last longer before the division planes are also orientated planar and form a tubular character. Stem cells do not differentiate, and hence, tubular outgrowths forming the secondary/tertiary rhizoids are missing (Løvlie, 1964, 1968). The two layers are filled with a lumen and joined at the margin, developing into an expanded band. The base of the primary rhizoid of the growing alga is not strong enough to hook the alga up with the substratum (Løvlie, 1964, 1968).

As the microflora is associated with the algae, it must be considered as part of *Ulva*'s environment, and thus, algal growth substances may be produced by epiphytic bacteria or fungi, rather than by *Ulva* itself (Goecke et al., 2010). Many studies have attempted to extract external compounds with growth regulatory activities motivated by the evidences that marine bacteria produce auxin- and cytokinin-like regulators (Schiewer, 1967; Maruyama et al., 1986, 1988, 1989), which seemed to be effective in ulvacean morphogenesis (Provasoli, 1958b; Fries and Aberg, 1978). This has already accumulated into a controversial discussion between Bradley (1991) and Evans and Trewavas (1991) concerning whether “*Plant hormones do have a role in controlling growth and development of algae*” and “*Is algal development controlled by plant-growth substances*,” summarized in the textbook “Seaweed Ecology and Physiology” (Lobban and Harrison, 1997). Several studies have looked at the effect of applying higher plant growth regulators (auxins and cytokinins). Auxin has been shown to promote algal growth or rhizoid formation in seaweeds, among them *Bryopsis*, *Caulerpa*, and *Ulva*, under culture conditions. Interestingly, a substratum of sand enriched with microorganisms was as effective in promoting algal development as the samples treated with phytohormone (Moos, 1974; Bradley, 1991; Evans and Trewavas, 1991; Lobban and Harrison, 1997) suggesting that bacteria playing an important role in algal development. Without the appropriate specific bacteria *Ulva* develops into a callus-like morphotype, sometimes described as an atypical but viable pincushion-like morphology composed of uniseriate branching filaments (e.g., Provasoli, 1958b; see Section The Symbiotic Nature of *Ulva* Growth—A Short History). These abnormal algal colonies can be partly (Bonneau, 1977; Provasoli and Pintner, 1980; Marshall et al., 2006; Singh et al., 2011; Spoerner et al., 2012; Vesty et al., 2015) or completely recovered (Spoerner et al., 2012) to their typical thallus by inoculation with appropriate marine bacteria or partly purified morphogenetic compounds (= morphogens). They govern the pattern of tissue development in the process of morphogenesis (Table 1). Working with axenic cultures (see Section Key Prerequisites Elucidating Cross-Kingdom Cross-Talk), the same specific bacteria induce the predisposed morphotype, such as wildtype or *slender*, respectively (Spoerner et al., 2012). Predisposition has also been observed prior to germ-cell production related to genetic control of cellular differentiation of the *bubble* like morphotype, which is another naturally occurring developmental mutant of *U. mutabilis* (Fjeld, 1972). In fact, bacteria are unable to endogenously govern the inherent program of morphogenesis of *Ulva*, but they are essential to induce growth, development, and morphogenesis.

**Table 1:** **Overview of studied interactions between Ulvales and associated bacteria related to bacteria induced morphogenesis (in chronological order)**.

**Algal Species**	**Source for the preparation of axenic cultures**	**Methodology to prepare axenic cultures**	**Experimental details of axenicity tests**	**Representative isolated morphogenesis-inducing bacteria (= MG active)**	**References**
*Ulva lactuca*	Small pieces of the thallus (1–5 mm^2^) were placed on antibiotic treated and melted agar.	Mixture of four antibiotics: penicillin G, chloramphenicol, neomycin, polymyxin B sulfate	Smaller strips of the axenic culture were inoculated in enriched seawater ASWIII or ASW8 for testing.	Bacteria were not isolated.	Provasoli, 1958b
*Enteromorpha linza*	Small pieces of the algae	Mixture of two antibiotics: penicillic acid, streptomycin sulfate	Test medium: ASP 6F + Glucose (Fries, 1963)	Bacteria were not isolated.	Berglund, 1969a,b
*Enteromorpha linza Enteromorpha compressa*	Same material was used by Berglund (1969a,b)	Mixture of two antibiotics: penicillin, streptomycin	Not reported	Bacteria were not isolated.	Fries, 1975
*Ulva lactuca*	“Swarmers” from axenic thallus	Dragged through agar, 15 antibiotics, 8 sulfa drugs, fungicide, Jodopax	Sterility test based on STP and ST3 media according to Tatewaki and Provasoli (1964) and by electron microscopy	Bacteria were not isolated.	Bonneau, 1977, 1978
*Ulva lactuca*	Axenically maintained strain Ulva-58 prepared by Provasoli (1958b) was used.	Mixture of four Antibiotics: penicillin G, chloramphenicol, neomycin, polymyxin B sulfate	Smaller strips of the axenic culture were inoculated in enriched seawater ASWIII or ASW8 for testing (Provasoli, 1958b).	Bacteria with MG were isolated, but not further characterized (Provasoli and Pintner, 1980).	Provasoli and Pintner, 1977, 1980
*Monostroma oxyspermum*	Motile germ cells: gametes or zoospores	Axenic cultures were obtained by repeatedly washing of motile stages with capillary pipettes or by washing thalli through the “dip and drag” treatment inside agar plates.	According the sterility test based on STP and ST3 media (Tatewaki and Provasoli, 1964)	Bacteria were not isolated (study with bialgal cultures).	Tatewaki, 1983
*Ulva pertusa*	Axenic female strain of *U. pertusa* (strain UP-203) was maintained aseptically.	Not reported	Platting on various agar media e.g., ST3, 2216 (Difco, USA), ESS_1_B_1_, ESS_1_B_2_ (Nakanishi and Saga, 1993)	– 1555 strains were isolated from 18 macroalgae, – 676 were MG active selected genera: *Cytophaga, Flavobacterium, Vibrio, Pseudomonas, Halomonas, Escherichia*.	Nakanishi et al., 1996, 1999
*Monostroma oxyspermum*	Unicellular strain MK-001 of *M. oxyspermum* in axenic culture	Not reported for *M. oxyspermum*	Not reported	– 1000 strains were isolated from macroalgae and corals, – 50 MG active strains belonged to the *Cytophaga-Flavobacterium-Bacteroides*-complex	Matsuo et al., 2003, 2005
*Ulva pertusa*	Motile germ cells were collected from *Ulva* strains.	For *Ulva*: germ cells were treated with antibiotics: penicillin G, streptomycin, erythromycin, kanamycin.		Thallusin producing strain: *Cytophaga* sp. (YM2-23) was isolated from *M. oxyspermum*.	
*Ulva conglobata*					
*Enteromorpha intestinalis*					
*Ulva linza*	Released zoospores were collected from axenic thallus strips.	Antibiotics: penicillin G, streptomycin, norfloxacin, kanamycin	Zoospores were spread on artificial algal agar medium	– 38 strains were isolated from three *Ulva* species, – 20 strains were MG active, selected strains: *Cellulophaga* sp. (UL16), *Cytophaga* sp. (UC19), *Psychrobacter* sp. (ULA5), *Pseudoalteromonas* sp. (UL34), *Shewanella* sp. (UL19).	Marshall et al., 2006
			Axenic calli were picked off after 35 days and cultivated in UCM (Stratmann et al., 1996).		
*Ulva fasciata*	Zoospores were collected from axenic thallus.	Chemical treatment with detergent and povidone-iodine followed by antibiotic treatment (Kumar et al., 1999; Reddy et al., 2006)	Axenicity of the algal culture was tested by incubating randomly selected algal tissue on nutrient and Zobell's agar medium (Zobell, 1941).	– 53 strains were isolated from different *Ulva*/*Gracilaria* species	Singh et al., 2011
				– 5 strains were MG active	
				*Bacillus flexus* (UL)	
				*Bacillus* sp. (GS)	
				*Bacillus* sp. (UL24)	
				*Bacillus licheniformis* (GC)	
				*Marinomonas* sp. (UF).	
*Ulva mutabilis*	Positively phototactic gametes were collected.	Axenic cultures were obtained by repeatedly washing of gametes *via* their strong positively phototactic response in capillary pipettes	Platting on marine broth agar plates (2216, Difco, USA) and by direct PCR of 16S rDNA in algal growth media	– 12 strains were isolated from *U. mutabilis*,	Spoerner et al., 2012
				– 4 strains were MG active	
				*Cytophaga* sp. (MS6)	
				*Roseobacter* sp.(MS2)	
				*Sulfitobacter* sp. (MS3)	
				*Halomonas* sp. (MS1).	
*Ulva linza*	Positively phototactic gametes were collected.	Axenic cultures were obtained by repeatedly washing of gametes *via* their strong positively phototactic response in capillary pipettes.	Platting on marine broth agar plates (2216, Difco, USA)	Cross-test with *Cytophaga* sp. (MS6) and *Roseobacter* sp. (MS2) originally isolated from *U. mutabilis* (Spoerner et al., 2012)	Vesty et al., 2015

Methodologies and representative isolated strains are listed, which were particularly tested or mentioned. The closest matching strain in Genbank/EMBL was usually given in the respective study; the accession number of the 16S rDNA analyses can be found in the cited publication. Bacteria were isolated from various macroalgae species in most studies.

Recent comprehensive reviews have examined the literature focusing on various aspects of microbial communications associated to macroalgae (Goecke et al., 2010; Friedrich, 2012; Egan et al., 2013, 2014; Mieszkin et al., 2013; Singh and Reddy, 2014). This review is particularly devoted to the multiple most intriguing ecophysiological effects of bacteria on the cell division/differentiation and morphogenesis of *Ulvales*. Addressing these multiple interactions, particularly the reception and transduction of signal molecules within the chemosphere of the symbionts, various disciplines are required, covering algae and developmental biology, microbiology, biochemistry, and analytical chemistry.

The interdisciplinary research is also reflected by Bradley's (1991) original critical remarks in demonstrating that the growth regulatory roles of hormones in macroalgae are still valid for the ongoing research of chemical compound-induced algal morphogenesis. Bradley's remarks can be summarized as follows (in excerpt):

- “The presence of the chemical compound should preferably be measured at the exact location in the plant.”
- “If the site of the hormone production can be excised, the response should stop.”
- “The response should (…) be restored with the addition of a chemical known to be an appropriate hormone.”
- “If the reacting system can be isolated from the plant (i.e., in vitro), then the effect should be the same as it was in vivo.”
- “The chemical should be shown to be present in similar cases (…) polarity, regeneration or apical dominance could be regulated to the same hormones (…).”
- “The chemical should show a specific response so that a clear relationship, a response and a hormone can be demonstrated.”
- “The effect of the chemical should be different in strains that have a specific mutation in the process that the hormone is controlling.”

Essential approaches and protocols are described necessary for the preparation of axenic algal tissues, bacteria isolation, co-cultivation experiments, and for bioassay-guided purification of morphogenetic compounds. The review highlights the achievements during the last eight decades and discusses novel research lines in analytical chemistry to decipher the cross-kingdom cross-talk between bacteria and macroalgae in laboratory and field studies.

## The symbiotic nature of *Ulva* growth—a short history

**Figure 4:**
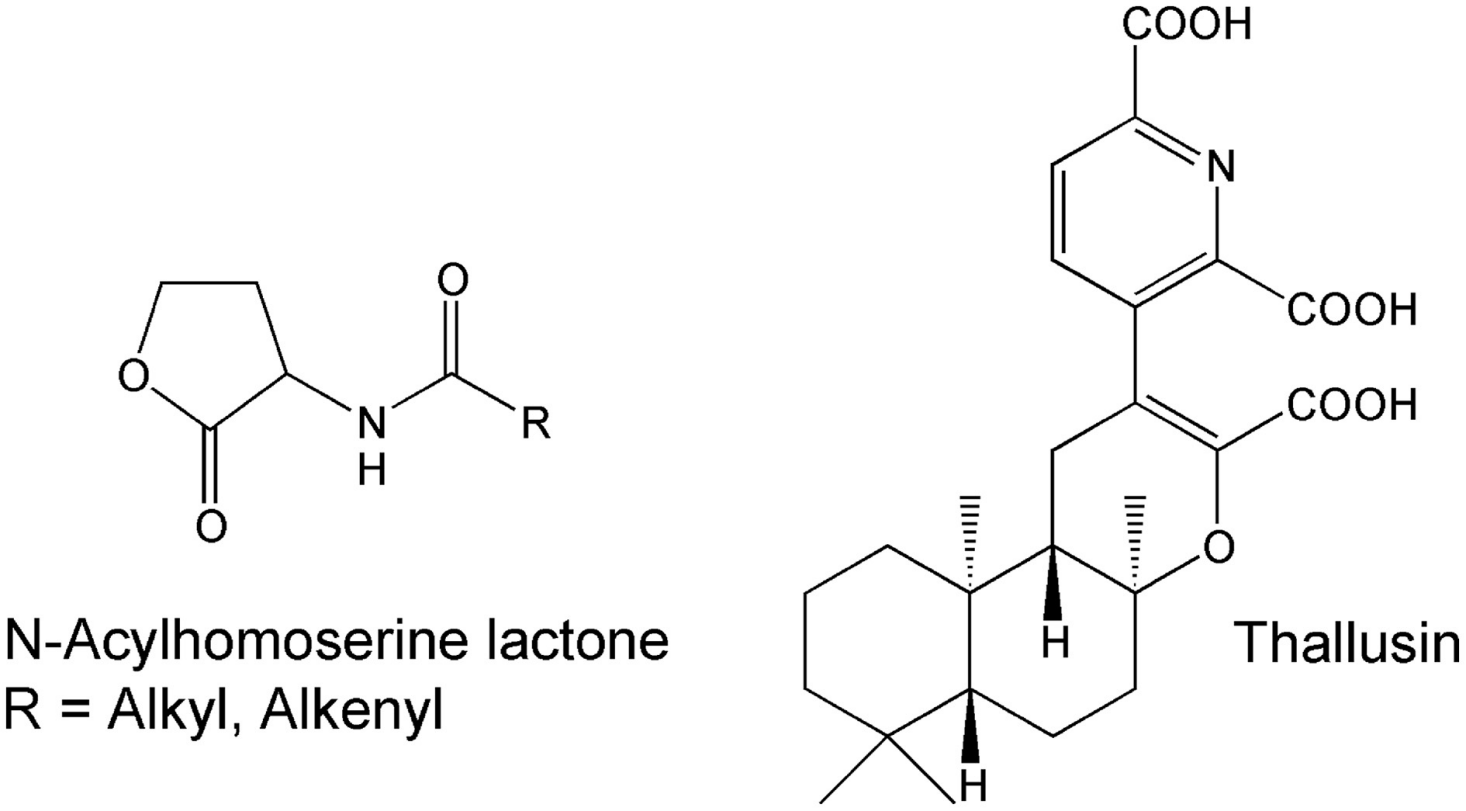
**Community-structuring molecules**. N-Acyl homoserine lactones (AHL) are a class of signaling molecules involved in bacterial quorum-sensing and thallusin is the first identified morphogenesis-inducing compound in macroalgae (Matsuo et al., 2005; Wheeler et al., 2006).

Cross-kingdom interactions between the *Ulva* spp. and its associated bacteria have been studied by several research groups. Table 1 illustrates their wide interest and versatile research carried out. The role of biotic interactions, such as allelopathic and chemotactic effects, which shape the assemblages of species in the algal environment is complex to study and made well-defined laboratory experiments along with intensive field studies necessary to prove the ecological relevance. A few bacterial infochemicals have been already demonstrated as being involved in the cross-kingdom cross-talk of Ulvales and its associated bacteria, including N-(3-oxododecanoyl)-homoserine lactone (AHL) for the settlement-modulation of zoospores and thallusin for induced algal morphogenesis (Figure 4). Besides the underlying mechanism and the metabolites involved, it is particularly intriguing, whether direct cell contact between bacteria and algae is necessary for their (symbiotic) interactions (see Section Key Prerequisites Elucidating Cross-Kingdom Cross-Talk).

Over the past six decades five species of *Ulva* have been studied extensively including (i) *U. lactuca*, (ii) *U. linza* (formerly *Enteromorpha linza*), (iii) *U. pertusa*, (iv) *U. fasciata*, and (v) *U. mutabilis* (Table 1). In addition to the distromatic sheets (“sea lettuces”) and monostromatic tubules (“enteromorpha”) of *Ulva*, the monostromatic *Gayralia oxyspermum* (synonym: *Monostroma oxyspermum*) was also well-investigated (Table 1). Morphogenesis-inducing extracts were isolated from various isolated bacteria belonging to the genera *Cytophaga*, *Flavobacterium*, *Caulobacter*, and *Pseudomonas* (Provasoli et al., 1977; Tatewaki, 1983). Matsuo et al. (2003, 2005) identified *Cytophaga* sp. (YM2-23) of the *Cytophaga-Flavobacterium-Bacteroides* complex, which excretes the morphogenesis-inducing substance thallusin and restores the foliaceous morphology of *M. oxyspermum*. The same factor also partially promotes the formation of distromatic thalli of *U. pertusa* and other *Ulva* species, highlighting the potentially important role of thallusin for the normal development of green macroalgae. Pure thallusin strongly induced the differentiation of *M. oxyspermum*, even at very low effective concentrations between 1 fg mL^−1^ and 1 ag mL^−1^ (Matsuo et al., 2005; Gao et al., 2006). Although thallusin can be obtained from bacterial cultivations, several total syntheses of thallusin and its analogs were performed to allow a detailed examination of thallusin's biological activity. Whereas, the compound originally synthesized (*ent*-thallusin) did not show any morphogenesis-inducing activity (Gao et al., 2006), the diametrically opposed compound ((±)-thallusin) was highly active, as were most of its analogs [e.g., the trimethyl ester (Nishizawa et al., 2007), although its activity decreased dramatically]. The free carboxylic residues and picolinic acid moiety seem to be thus indispensable for strong morphogenesis-inducing activity at low concentrations, and the diterpene skeleton plays an important role inducing differentiation processes in *M. oxyspermum* (Nishizawa et al., 2007).

The research on the bacteria-induced morphogenesis in *Ulva* started with the fundamental experiments of Provasoli (1958b), who tested the hypothesis “*if Ulva, when bacteria-free, would grow in mineral media or if it would require organic factors*.” Provasoli (1958a,b) failed to obtain a typical foliaceous thallus in media similar to “Erdschreiber” without bacteria, but, in trying to obtain it, he found that *Ulva* germlings responded to plant phytohormones. For the first time, an attempt was made to culture *Ulva* axenically starting with thalli treated with antibiotics (Table 1). Upon sporogenesis, the motile zoospores were collected and further cultivated in sterile artificial seawater media (ASW8): As young germlings developed into a callus rather than in a complete thallus, Provasoli (1958b) concluded that morphogenetic regulators are missing. Indeed, plant hormones such as kinetin and auxin (Provasoli, 1958b), or phenylacetic acids and its hydroxyl derivatives (Fries and Aberg, 1978) favored significantly the elongation of the germlings and/or morphogenesis of *Ulva*. However, the observed effects were still questionable (Buggeln, 1981), because the plant growth regulators did not induce the complete normal morphology (Provasoli and Carlucci, 1974). In any case, the effects of the tested compounds were different to the growth-stimulating activity of e.g., vitamins in callus-like axenic cultures of *Ulva* (Fries and Aberg, 1978). Therefore, Provasoli and Pintner (1980) tested the effect of isolated, but not taxonomically described bacteria, on *U. lactuca.* Several polymorphisms were found in the bacterized growth media of *Ulva*, but no combination could recover the development of a typical expanded foliose thallus. Moreover, bacteria filtrates were inactive, indicating that symbiotic/syntrophic growth is necessary in this genus (Provasoli and Pintner, 1980) or even the direct cell contact of bacteria and alga (Nakanishi et al., 1996, 1999). In any case, the research on the genus *Ulva* revealed species-specific response to the associated bacteria.

Marshall et al. (2006) screened a collection of 38 unique isolates for their effects on the growth and morphology of axenic plantlets cultivated from released zoospores. Hereof, 20 unique marine epiphytic bacteria were stimulating the development of axenic *U. linza* cell platelets (treated with antibiotics) into numerous tubular structures to various extents. Plant morphology was assessed on a semi-quantitative scale based on the number and state of development of tubules extending from the central callus of each plant (Table 1). However, no completely normal development of these algae into mature thalli in the presence of only one of the bacterial strains tested was documented (Marshall et al., 2006). These results have already indicated synergistic effects of bacteria on the thallus development, which were later found in *U. mutabilis* and *U. linza* (Spoerner et al., 2012; Vesty et al., 2015).

Only a few out of 35 bacterial species obtained from *Ulva fasciata*, in particular *Marinomonas* sp., were able to induce morphogenesis and growth of zoospores derived from axenic thalli (Singh et al., 2011). Differing from the other studies of, for example, Spoerner et al. (2012) and Marshall et al. (2006), the axenic cultures of *U. fasciata* still divided directionally and developed into thalli of e.g., 146 μm^2^, suggesting that other factors rather than those provided by the isolated bacteria were supporting growth and directing cell division.

**Figure 5:**
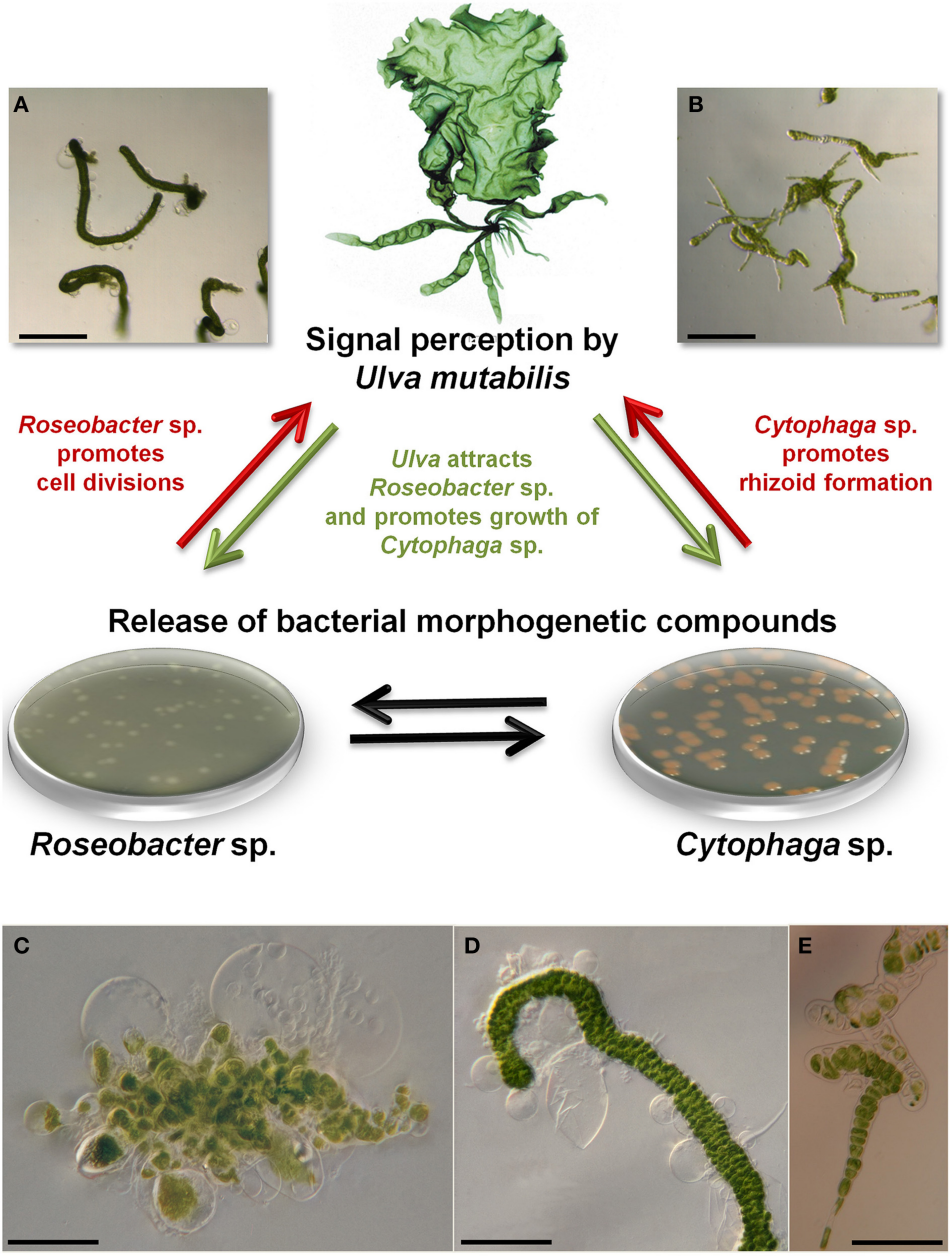
**Tripartite community and representative morphotypes**. Tripartite community of *U. mutabilis* with proposed essential interactions for standardized experimental set-ups. A combination of *Roseobacter* sp. (MS2) and *Cytophaga* sp. (MS6) excreting morphogenetic substances recover growth and morphogenesis of the wildtype (shown in the top) and the mutant slender of *U. mutabilis*: *Roseobacter* sp. promotes cell division (**A**,**D**: scale bars = 1 mm and 500 μm) and *Cytophaga* sp. promotes rhizoid and cell wall formation (**B**,**E**: scale bars = 1 mm and 100 μm). Strictly axenic cultures develop into a callus like morphotype consisting of undifferentiated cells without normal cell walls (**C**: scale bar = 100 μm). The aberrant axenic morphotype shows unusual cell wall protrusions and no differentiated rhizoid cells. **(D)** When *Roseobacter* sp. was added to axenic cultures, dark green germlings appeared with cell walls covered by typical bubble-like structures. **(E)** When *Cytophaga* sp. was added to axenic cultures, short rows with partly degenerated cells, but normal cell walls, were observed (Spoerner et al., 2012). Images were provided by Jan Grüneberg (University Jena, Germany).

Axenic *U. mutabilis* cultures derived from purified phototactic gametes develop into a callus-like morphotype, where only cell divisions with colorless protrusions from the exterior cell wall are promoted (Figure 5C). In this study, the complete morphogenesis was recovered either by the combination of the two bacterial strains *Roseobacter* sp. and *Cytophaga* sp., or by morphogenetic compounds extracted from the bacterial supernatant (Spoerner et al., 2012). *Roseobacter* sp. and *Cytophaga* sp. fulfill complementary tasks: *Roseobacter* sp. (MS2) induces cell division similar to a cytokinin (Figures 5A,D), whereas the *Cytophaga* sp. (MS6) promotes, similar to auxin, a viable basal stem cell and primary rhizoid cells in addition to the induction of the proper cell wall formation (Figures 5B,E). Moreover, the *Roseobacter* species exhibits a specific chemotactic affinity to the rhizoid cells of *U. mutabilis* and seems to cooperate with the *Cytophaga* strain and the alga by chemical communication, forming a symbiotic unique tripartite community (see Section Interactions within a Tripartite Community and Biofilm Formation). Spoerner et al. (2012) further demonstrated that *Cytophaga* sp. releases at least two different morphogens working synergistically, which were partly purified by size-exclusion chromatography.

According to Bradley's (1991) postulations (see Section Introduction), the response would stop and the germling/thallus will develop cell walls with spherical structures again, if the morphogen producing bacteria has been removed, which can be facilitated experimentally by a two-chamber set up (see Section Survey of Diffusible Compounds: Two-Chamber Cultivation of Bacteria and *Ulva*). The still unknown bacterial compounds, which have a morphogenetic effect on *Ulva*, are hard to identify due to their very low concentration in the medium. In any case, known phytohormones could not replace the bacteria in *U. mutabilis* (Spoerner et al., 2012) or in *U. linza* (Fries, 1975).

Zoospores of *U. compressa* treated with penicillin and streptomycin developed into germlings, supposed to be axenic, harboring colorless protrusions from the exterior cell walls with spheres of cytoplasm (Fries, 1975). Interestingly, the same incomplete morphotype was found in *U. mutabilis*, when axenic gametes were inoculated with *Roseobacter* sp. or a partly purified *Roseobacter* factor (Figure 5A) (Spoerner et al., 2012). It is thus tempting to speculate that the microflora of *U. compressa* was only partly eliminated, which often leads to an incomplete morphology (Provasoli and Pintner, 1980; Spoerner et al., 2012). Cultures of *U. compressa* were potentially contaminated with a *Roseobacter* strain. This is even more likely, as species of the *Roseobacter* clade possess a considerably low susceptibility to antibiotics (minimal inhibitory concentration is often >100 μg mL^−1^ (Piekarski et al., 2009). Antibiotics at pretty low concentration (i.e., <10 μg mL^−1^; Matsuo et al., 2003) have been used to treat various algal culture media in several studies (Table 1). Therefore, a proper axenicity test is always particularly valid (see Section Axenic Algal Cultures).

The underlying mechanisms that induce cell fate during algal development remain unknown. Therefore, a combined experimental and modeling study on cross-talk is important for elucidating the complexity of the bacteria-algae interactions, which can be reduced to a *tripartite* symbiosis in the case of *U. mutabilis* or a *bipartite* relationship in *M. oxyspermum*. From a critical point of view, variations in all these studies described might be (i) due to a lack of standardization or lack of axenicity of the original *Ulva* cultures, (ii) due to a biased approach for bacteria-isolation, and (iii) due to the loss of the capability for morphogen production after cultivation of isolated bacteria in marine broth media.

## Key prerequisites elucidating cross-kingdom cross-talk

Before studying the cross-kingdom cross-talk, several essential prerequisites have to be fulfilled in order to provide a robust, reliable, and repeatable experimental set-up. This includes the preparation of axenic cultures, the isolation and cultivation of bacteria, considering that most of them might be not cultivable (without the hosts/symbionts), and proper bioassays to determine the morphogenetic compounds by fractionated purification (Matsuo et al., 2005; Spoerner et al., 2012) or comparative metabolomics (Fiehn, 2001; Vidoudez and Pohnert, 2012; Gillard et al., 2013). The lack of standardization of the experiments involves contradictory observations made by different research groups rather than the determination of species-specific interactions between *Ulva* and its associated bacteria. In this section, several key prerequisites are suggested to obtain trustable data from bioassays:

- Axenic algal cultures supplemented with specific bacteria should be used rather than wild algae or uncontrolled bacterized cultures. The axenicity should be tested before and during the bioassays.
- Successful isolation/cultivation of morphogenesis-inducing bacteria might depend on the addition of algal compounds in bacterial growth medium.
- Two-chamber cultivation systems should be used to prove cross-kingdom cross-talk via diffusible water-borne compounds.

### Axenic algal cultures

The main challenge is how to get rid of the accompanying microflora of the algae and its mucilaginous layer. Whereas, the usage of axenic cultures is quite common for microalgae, this is limited for the study of marine macroalgae. Axenic seaweed cultures might be obtained either by the addition of antibiotics to the growth medium or by a combination of antibiotic application and subsequent isolation of reproductive cells (Provasoli, 1958a; Andersen, 2005).

Using antibiotics, it is not always possible to remove all of the microorganisms associated with *Ulva*. Yeast and several marine bacteria are unaffected by antibiotics and diatoms have to be eliminated by GeOH_4_ or silica-free growth media (Andersen, 2005). It must be also cautioned that antibiotics might affect the growth of *Ulva* (Andersen, 2005). The success of antibiotics depends very much on the microbial flora being sensitive to the applied substances (Tatewaki and Provasoli, 1964). Agar plates can be effective for cleaning, if filaments of the thallus are dipped several times into (the antibiotic containing) agar with a stiff glass needle. This “dip and drag” technique is based on the removal or cleansing of the slimy surface of the thallus by friction against the agar (Tatewaki and Provasoli, 1964). Another successful approach for the surface sterilization of *Bryopsis* plants was elaborated by Hollants (2012), but has not yet been applied to the order Ulvales. *Bryopsis* thalli were treated with a combination of proteinase K and the bactericidal cleanser Umonium Master in CTAB lysis buffer and hence effectively sterilized. Amplified 16S rDNA only resulted from endophytic bacteria (Hollants et al., 2011; Hollants, 2012). Protoplasts were also successfully facilitated as feedstock for axenic cultures (Reddy et al., 1989). Unialgal material was firstly treated with KI solution and, subsequently, treated with antibiotics before thallus material was incubated in a mixture of commercially available cellulose and an enzymatic crude extract from an abalone. The regenerated protoplast can be used for further tests of the bacterial morphogenetic compounds, although those cells might not be longer omnipotent and are thus not necessarily representative for studies of cross-kingdom interactions and epigenetic mechanisms to control variations in morphotypes. In *U. mutabilis*, single isolated protoplasts prepared from blade cells develop only into hollow spheres composed of blade cells only. They are not able to develop directly into complete plants, in opposition to germ cells or isolated stem and rhizoid cells (Fjeld and Løvlie, 1976).

**Figure 6:**
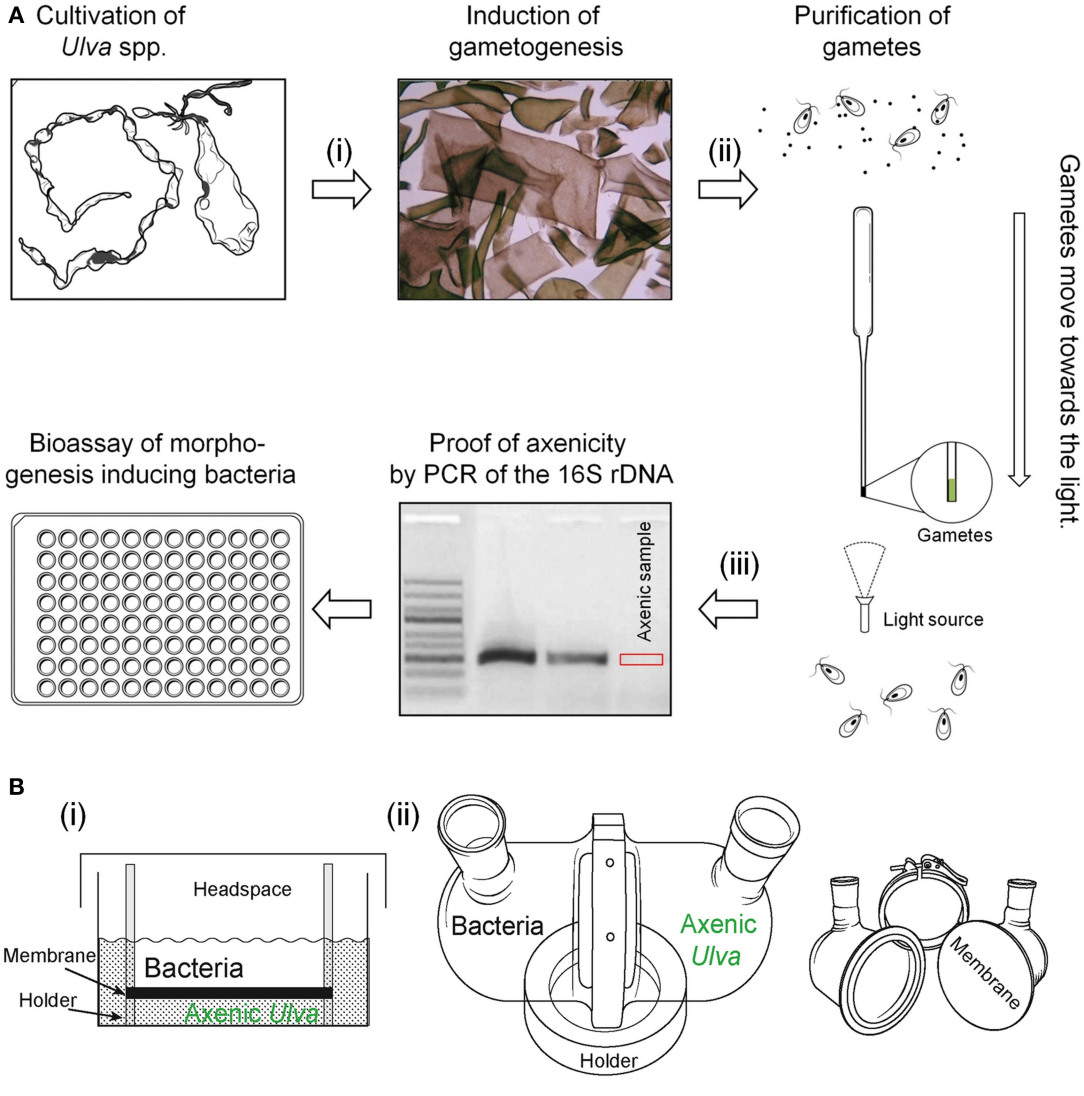
**Purification of gametes and two-chamber systems. (A)** Three steps to a robust multiwell-based bioassay for screening the potential algal morphogenesis-inducing bacteria: Upon induction of gametogenesis (i) by removal of sporulation inhibitors through mincing and washing of mature thalli, (ii) gametes are purified through their phototactic movement towards light. Concentrated gametes can be collected from the top of the capillary pipette, and (iii) subjected to the axenicity test by PCR testing of contaminants in seawater media. Gel image was used and modified with permission from Spoerner et al. (2012; Copyright©2012, Wiley). **(B)** Set-ups of two-chamber reactors for studying diffusible morphogenetic or allelopathic compounds are presented: (i) Simplified two-chamber system suitable for multiwell plates used by Spoerner et al. (2012) using a 0.02 mm Anopore™ Nunc Filter. (ii) Assembled up-scaled two-chamber system (Paul, 2012) and the disassembled setup with all parts required. Each chamber, separated by a 0.22 mm hydrophilic polyvinylidene fluoride (PVDF) membrane filter, can be filled with 400 mL culture medium. Sterile filling and sampling of large volume is feasible.

An elegant way to separate germ cells from their accompanying bacteria can be performed by the movement of phototactic gametes through glass capillary pipettes (Tatewaki and Provasoli, 1964; Stratmann et al., 1996; Spoerner et al., 2012). Gametes assemble at the brightest spot of the sporulation dish (Figure 6A). A dense solution with gametes can be collected easily and be loaded onto a horizontally laying capillary filled with sterile artificial seawater (under strictly sterile conditions!). The majority of gametes move horizontally to the top of the pipette, where they can be again collected in few microliters of sterile seawater before the procedure has to be repeated two times. The gametes outcompete the accompanying microflora on a total length of about 0.5 m (i.e., three washing steps through Pasteur pipettes) (Figure 6A).

Whatever methodology has been used, axenicity has to be tested by a reliable test, which might be a serious pitfall of several studies. Taking into account that a concentration of 1 ag mL^−1^ of thallusin can induce the morphogenesis in *M. oxyspermum* sufficiently (Matsuo et al., 2005), even the smallest bacterial contamination will interfere with the screening for potentially morphogenesis-inducing bacteria. The most common traditional “sterility test” uses two general media (ST3 and STP) for marine bacteria detailed described by Tatewaki and Provasoli (1964). Sterility test media are inoculated with the sample in liquid and in agar form and inspected microscopically over 1 month for bacterial growth. However, incubating thalli disks, zoospores or gametes treated with antibiotics on various agar plates or liquid media is an insufficient approach, as only cultivable bacteria will be observed. Only a small fraction (<1%) of microorganisms grow in standard culture media or on agar plates (Amann et al., 1995). Overall, additional tests have to be conducted in order to prove axenicity, for example, by 4′,6-diamidino-2-phenylindole (DAPI) staining or fluorescence *in situ* hybridization (FISH). For rapid testing of axenicity, Spoerner et al. (2012) recommended to amplify the highly conserved bacterial 16S rDNA *via* polymerase chain reaction (PCR) with specific primer pairs (Figure 6A). Total bacterial DNA can be isolated upon filtration of the supernatant through filter units and subsequent lysozyme and proteinase K treatment. Alternatively, PCR should be used for the direct detection of bacteria in culture media without the requirement for isolation ensuring before that matrix effects do not inhibit the PCR (Spoerner et al., 2012). The improved primer pairs for the 16S rDNA of marine bacteria might be applied (Sanchez et al., 2007).

### Complex microbial communities: isolation and cultivation of bacteria associated with *Ulva*

A bioassay-driven approach demands viable strains in culture media. Up to 1500 strains were isolated by several research groups during single campaigns searching for morphogenesis inducing bacteria in Ulvales. Bacteria associated with seaweeds were isolated from a small piece of freshly collected alga, washed in sterile water and, finally, plated on agar plates, for example, marine broth 2216 (Difco, USA) supplemented with agar (Matsuo et al., 2003). Other protocols suggested swabbing the surface of the alga in order to transfer bacteria on an agar plate. In any case, each of the methodologies is highly biased as it depends on the selected media promoting bacterial growth. To circumvent biases inherent to bacterial cultivation from environmental samples, molecular techniques such as denaturating or temperature gradient gel electrophoresis have very often been used to profile complex communities in a cultivation-independent manner (Muyzer et al., 1993; Ferris et al., 1996; Lachnit et al., 2009). FISH can be used with e.g., specific 16S rRNA probes to detect taxonomic groups at the genus level and is extensively used to detect unknown and uncultivable bacteria (Amann et al., 1995).

*Ulva*-associated bacterial communities described with cultivation-dependent and -independent methods usually show little correspondence at the approximate species (operational taxonomic unit). Studies have shown that the majority of cultivatable marine bacteria belong, in general, to the α-Proteobacteria or γ-Proteobacteria, and that is also true for *Ulva* (Hagstrom et al., 2000; Tujula et al., 2010). However, strains relevant for the morphogenesis may not yet have been identified (Mieszkin et al., 2013), because they are not cultivable in standard culture media. In particular, species of the Cytophaga/Flavobacteria group are often hard to culture or even grow only in the presence of their symbionts (Amann et al., 1995; Gómez-Pereira et al., 2012; Spoerner et al., 2012). As several groups have already reported syntrophic or mutualistic interactions between algae and bacteria (Goecke et al., 2010), growth media should be improved by supplementing them with allelopathic substances extracted from *Ulva* or the supernatant of axenic algal cultures.

### Survey of diffusible compounds: two-chamber cultivation of bacteria and *Ulva*

Whether morphogenesis is only mediated by compounds released in the chemosphere of the symbionts or if direct physical cell contact is necessary, as proposed for certain *Ulva* species, is still under discussion. The fact that filtrates of bacterial cultures did not change the morphology of axenic cultures in some studies implied that intimidate or reciprocal relationship is necessary in e.g., *U. lactuca*. However, bacteria of morphogenetic activity can also lose this function when grown in artificial organic media without *Ulva* and regain it by co-cultivation with the alga (Provasoli and Pintner, 1980). To address this important question, Nakanishi et al. (1996) isolated a large number of marine bacteria species which stimulated algal development of axenic *U. pertusa* in a mixed culture. In fact, when axenic germlings and morphogenesis-inducing bacteria were inoculated in two chambers, separated by a nucleopore filter, no effect on the morphogenesis was observed suggesting that direct contact between *U. pertusa* and active bacterial strains is necessary (Nakanishi et al., 1996). In contrast, cell-free extract can partly induce the recovery of the morphogenesis of axenic *Monostroma* species (Provasoli and Pintner, 1964; Tatewaki, 1983). The addition of synthesized thallusin (Figure 4) to bacteria-free cultures of *Monostroma* (and to some extent of *U. pertusa*), proved finally that bacteria can be removed, if thallusin is continuously supplied (Matsuo et al., 2005). Diffusible morphogenetic substances released by bacteria were also found and partly purified by Spoerner et al. (2012) (Figure 6B). Axenic gametes can thus be separated by a Nunc Anopore™ (Nunc, Denmark) membrane from active bacteria. All necessary compounds to recover the algal morphogenesis were released into the water, so that no direct cell-cell contact was necessary. The Nunc Anopore™ filter sets allow the screening of various strains in multiwell plates for a lot of small bioassays (and different treatments) at the same time. For metabolite profiling and sampling during algal growth, larger amounts of medium are necessary for solid phase extraction (SPE) and compound isolation.

Paul et al. (2013) introduced a newly designed two-chamber device that allows culturing of lager amounts of algae and bacteria, which are physically separated, but can exchange dissolved or colloidal chemical signals. Identical growth conditions for both partners and high metabolite diffusion rates between the culturing chambers are ensured. This experimental set-up should be considered for further scaled-up two-chamber experiments with *Ulva* germlings and bacteria (Figure 6B, Paul et al., 2013). Similar diffusion growth chambers were also successfully applied for the cultivation of marine sponge-associated bacteria (Steinert et al., 2014).

## Approaching the molecular complexity of the algal-bacteria partnership

### Chemical analysis: from bioassay-guided fractionation to exo-metabolomics

The interactions between algae and bacteria depend strongly on chemical stimuli. These chemical cues are called infochemicals (Dicke et al., 1990) and their identification is essential to the understanding of signal-mediated cross-kingdom interactions. Because of land drainage, microbial growth is favored and might provide the necessary morphogenetic regulators essential for the growth of seaweeds. However, the dilution of the concentration and fluctuations of their level could control the speed and size of green tides in the coastal zone. Provasoli (1958b) pointed out: “*To resolve these issues, not only are extensive pure culture studies needed but also convenient sensitive methods for assaying plant hormones in sea water.*”

The chemosphere as a part of the biocoenose where the organisms interact with each other via infochemicals (Wichard et al., 2011; Alsufyani, 2014) can be investigated within the tripartite community of *U. mutabilis* in its most simplistic variation (Figure 5). Within this chemosphere, algal-associated bacteria produce signal molecules, toxins, allelopathic compounds, and morphogens, and provide nutrients and quorum-sensing (QS) signals interfering with the settlement of spores (Dobretsov and Qian, 2002; Croft et al., 2005; Weinberger et al., 2007; Goecke et al., 2010). To unravel the chemosphere, two major approaches should be followed in parallel for the further identification of morphogenetic compounds and metabolites which control life cycle and development: (i) A bioassay-guided fractionation and subsequent structure elucidation upon SPE of active compounds. This approach can be sufficiently supported by (ii) an unbiased monitoring of the exo-metabolome based on analyses of extracts using gas and liquid chromatography coupled to a mass spectrometer. A promising approach might be the survey of exo-metabolite profiles during the season or pre- and post-bloom events of *Ulva*. Algal growth phases can be studied under standardized culture conditions considering specific treatments such as e.g., low/high iron concentrations or changing bacterial communities. (Exo-)metabolomics would give a comprehensive view of waterborne organic substances released into the water body within the whole habitat or by the specifically studied community in the laboratory, respectively. Data analyzed by principal component analysis, as well by supervised discriminant function analysis, and subsequent validation by leave-one-out cross-validation will reveal specific biomarkers for a specific change in environmental conditions if they can be clearly defined as a e.g., pre- and post-bloom situation or intertidal events (Anderson and Robinson, 2003; Anderson and Willis, 2003). Alsufyani (2014) has recently shown that such biomarkers derived by exo-metabolite profiling are a valuable tool to predict changes in life cycle or to indicate potential contaminants in laboratory cultures. If signals only occur in the exo-metabolome of specific events, their role can be further pursued in a bioassay-based approach (Alsufyani, 2014). Finally, exo-metabolomic profiling should be supported by data employed in field experiments to validate the ecological role of the metabolites identified along with gene expression studies.

While a great emphasis has been placed on global metabolomic analysis in all fields of life sciences (Fiehn, 2001), analyses of specific subsets of compounds addressing a specific scientific question are still underdeveloped (targeted metabolomics) e.g., regarding detoxification and homeostasis of metals (essential vs. non-essential metals) in *Ulva*. For example, recruitment of essential metals can be facilitated by targeted analysis of metallophores, which are chelators of metal cations and oxoions (Deicke et al., 2013, 2014). The acquisition of trace metals in nitrogen fixing habitats or under iron-deficient conditions are of particular interest (Reid et al., 1993; Keshtacherliebson et al., 1995; Amin et al., 2007; Zhang et al., 2015). The presumably concerted action of heavy metal detoxification by *Ulva* (Wang and Dei, 1999; Villares et al., 2001) and its associated microorganisms along with bioremediation processes need to be elucidated by metal isotope coded profiling of metallophores in future studies (Deicke et al., 2014).

### Field experiments: eavesdropping cross-talk in a natural environment

Several laboratory studies have been conducted to understand the chemical communication of host-algal interactions (Goecke et al., 2010), but the ecological significance of these observations has not yet been supported by field experiments. Whereas, the microbiome of *Ulva*, collected from various sampling sites, was investigated intensively at several sampling sites around the world, chemical analyses of metabolites released into the water body are still scarce. However, first chemical analysis by ultrahigh pressure liquid chromatography coupled to a time-of-flight mass spectrometer (LC-ToF) revealed significant biomarkers, which describe the best specific environmental situations, such as tidal pools occupied by various seaweeds and sea grasses (Alsufyani, 2014). Certainly, it is tempting to search for morphogenesis-inducing compounds in the “noise of the ocean” using an explorative direct metabolic approach (Prince and Pohnert, 2010), however, the biological stimuli-responsive-concentration of the morphogenetic regulators is very low (= below the detection limit of mass spectrometric analysis even after extensive SPE), so that bioassay-guided approaches have to be taken into account again. Using standardized bioassays, morphogenetic activity could be undoubtedly determined in sterile-filtered seawater from the lagoon's tidal pools in the Ria Formosa (Faro, Portugal). The relative concentration was estimated by dilution series indicating very low concentrations, but high biological activity of both the *Roseobacter*- and *Cytophaga*-factors (Grueneberg et al., personal communication and Wichard et al., 2011). In order to decipher the (bacterial) origin of the morphogenesis-inducing activity in the lagoon's seawater, bacteria have to be isolated from both the waterbody and the algal surface to perform bioassays. All these experiments will be built on cultivation-based studies.

Metagenomics has emerged as a powerful sequence-based tool for describing the composition of natural microbial communities (Friedrich, 2012). This approach can be used to analyze the microbiome regardless whether the bacteria are cultivable or not, and will be very complementary to the chemical analysis of the chemosphere. Metagenomics is based on the genomic analysis of microbial DNA that is extracted directly from environmental samples and is, hence, an unbiased methodology that unlocks the diversity of bacteria in seawater. Most importantly, DNA has to be selectively extracted without any contamination from the host and can be used for clone libraries and metagenomic sequencing (Burke et al., 2011a). Further studies should aim at a holistic approach integrating metabolomics, transcriptomics, and sequence-based microbial community composition studies of *Ulva*'*s* chemosphere, where the cross-talk *via* infochemicals happens. A recent study has already successfully integrated the metabolomic and transcriptomic data of a brown macroalga (Ritter et al., 2014). This approach has far-reaching impacts on ongoing metabolomic studies of algal-bacterial interactions in changing environments (Dittami et al., 2014).

## *Ulva* gets what it needs

There is accumulating evidence that *Ulva*'s microbiome is defined stochastically where algal surface provides a niche for microorganisms carrying specific functional genes rather than belonging to specific microbial taxonomic entities (Burke et al., 2011a). Contrarily, very specific algal-bacteria interactions were described which implied host-specific microbial communities on macroalgae (Wheeler et al., 2006; Spoerner et al., 2012).

### Detection of quorum-sensing molecules by algal zoospores

QS is one of the best investigated processes of bacterial cell-cell communication. It is a density-dependent process that involves the production of extracellular, diffusible signal molecules that coordinate gene expression. A fascinating observation revealed that zoospores of *U. linza* are attracted to bacterial AHLs: this is ruled by chemokinesis rather than by chemotactic behavior, as zoospores do not swim directly towards the source (= bacterial biofilm) of AHLs. Indeed, when AHLs are detected by the zoospores, swimming speed decreases dramatically and tumbling starts, which results in an accumulation of zoospores at the site of AHL production (Tait et al., 2005). Background color and the surface chemistry contribute additionally to the settlement and germination success (Chaudhury et al., 2006; Finlay et al., 2008). The chemo-response to AHLs was dependent on the moiety of the acyl side chain, with N-(3-oxododecanoyl)-homoserine lactone being the most effective signal molecule (Wheeler et al., 2006). Although the AHLs have not yet been determined in natural samples, there is laboratory evidence proving the physiological effect of AHLs: They cause an influx of calcium and it is postulated that the reduction in swimming speed occurs through calcium-dependent modulation of the flagellar beat pattern. As this was the first observation of a calcium-dependent response to the bacterial AHL signal (Joint et al., 2000, 2002; Wheeler et al., 2006), the zoospores bacteria communication is an intriguing model for eavesdropping on cross-kingdom cross-talk.

The facility of a genetic system for *Ulva* will further help to unravel the communication (Wichard et al., 2015). The characterization of the signal transduction pathway in *Ulva* by genetic complementation of AHL-insensitive algal mutants, for instance, may identify the respective signaling pathways. However, the methodologies up to now are only available to address this question from the bacterial perspective (Piekarski et al., 2009).

Interestingly, the zoospore germination and growth of young germlings was improved in *U. linza* when AHL-producing strains, such as the *Sulfitobacter* spp. (376), were transformed with the AHL lactonase gene *aiiA* to generate AHL-deficient variants (Twigg et al., 2014). This revealed that the AHLs, which are released by *Sulfitobacter* spp. or the bacterial products they control, have a negative impact on germination. Nevertheless, strains of the *Roseobacter*/*Sulfitobacter* clade are essential for the growth and morphogenesis of *U. mutabilis* and *U. linza* (Spoerner et al., 2012; Vesty et al., 2015). Future work has to provide additional insight into the foundational assumptions of QS and relate laboratory discoveries to natural ecosystems (Schuster et al., 2013).

### Interactions within a tripartite community and biofilm formation

**Figure 7:**
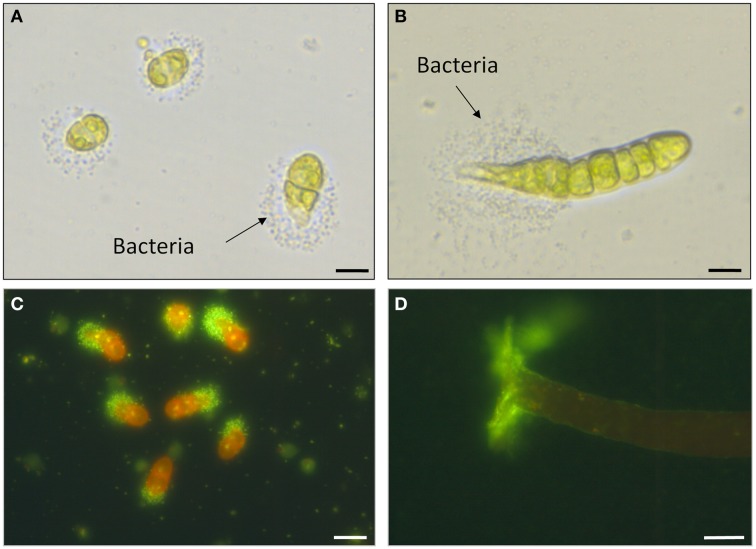
**Biofilm formation and potential cross-kingdom interactions. (A–D)** Biofilm formation upon inoculation of axenic *U. mutabilis* (wildtype) gametes with *Roseobacter* sp. (MS2) and *Cytophaga* sp. (MS6). **(A)** Two-cell stadium of *U. mutabilis* with the settlement of *Roseobacter* sp. nearby on the pole of the germling as observed by Spoerner et al. (2012). **(B)** Eight-cell stadium showing the expression of the primary rhizoid. **(C)** Same as **(A)**, but stained with SYBR® Gold Nucleic Acid (Life Technologies, USA) highlighting the bacteria at one pole of the young germling (**A–C**: scale bars = 20 μm). **(D)** Lateral view of a 5-week-old alga and SYBR® Gold Nucleic Acid stained biofilm of *Roseobacter* sp. (Scale bar = 100 μm). Images were provided by Jan Grüneberg (University Jena, Germany).

Interactions between microbial biofilms and marine-fouling algae were recently nicely reviewed (Mieszkin et al., 2013). Briefly, various isolated bacterial strains stimulated the settlement of the negative-phototactic zoospores, including *Vibrio*, *Shewanella*, and *Pseudomonas* strains that initiate the biofilm formation (Patel et al., 2003; Marshall et al., 2006). Conclusively, the effect on the settlement is species-specific rather than taxon-specific (Patel et al., 2003). The same was shown for the cell division-inducing morphogenetic compound (*Roseobacter* factor) which can be produced and released by various species (Spoerner et al., 2012). This raises the intriguing question whether there are strains that can contribute to both settlement and morphogenesis (Mieszkin et al., 2013). In fact, Mieszkin et al. (2013) have already pointed out that *“those bacteria that enhance spore settlement are not necessarily the same as those that initiate normal morphology and/or growth of the alga*.” *Vibrio* species release AHLs and contribute to the settlement of zoospores, but do not produce morphogenetic compounds (Marshall et al., 2006). However, it seems that the Cytophaga-Flavobacterium-Bacteroides group and the *Roseobacter* clade are particular important as they might combine potentially both ecophysiological functions. Members of both groups are able to release AHLs (Tait et al., 2005; Wagner-Dobler et al., 2005) and morphogenetic compounds (Spoerner et al., 2012). A combined approach of chemical analysis for both AHLs and morphogens is needed along with bioassays testing the activity for chemokinesis and induction of algal morphogenesis. The tripartite community of *U. mutabilis, Cytophaga* sp., and *Roseobacter* sp. might be an ideal system to investigate (Figure 5) (i) the chemotactic attraction of bacteria (Figure 7), (ii) the QS systems not only between bacteria, but also between bacteria and the host, and (iii) the interaction between the macroalga and opportunistic bacteria/pathogens.

**Figure 8:**
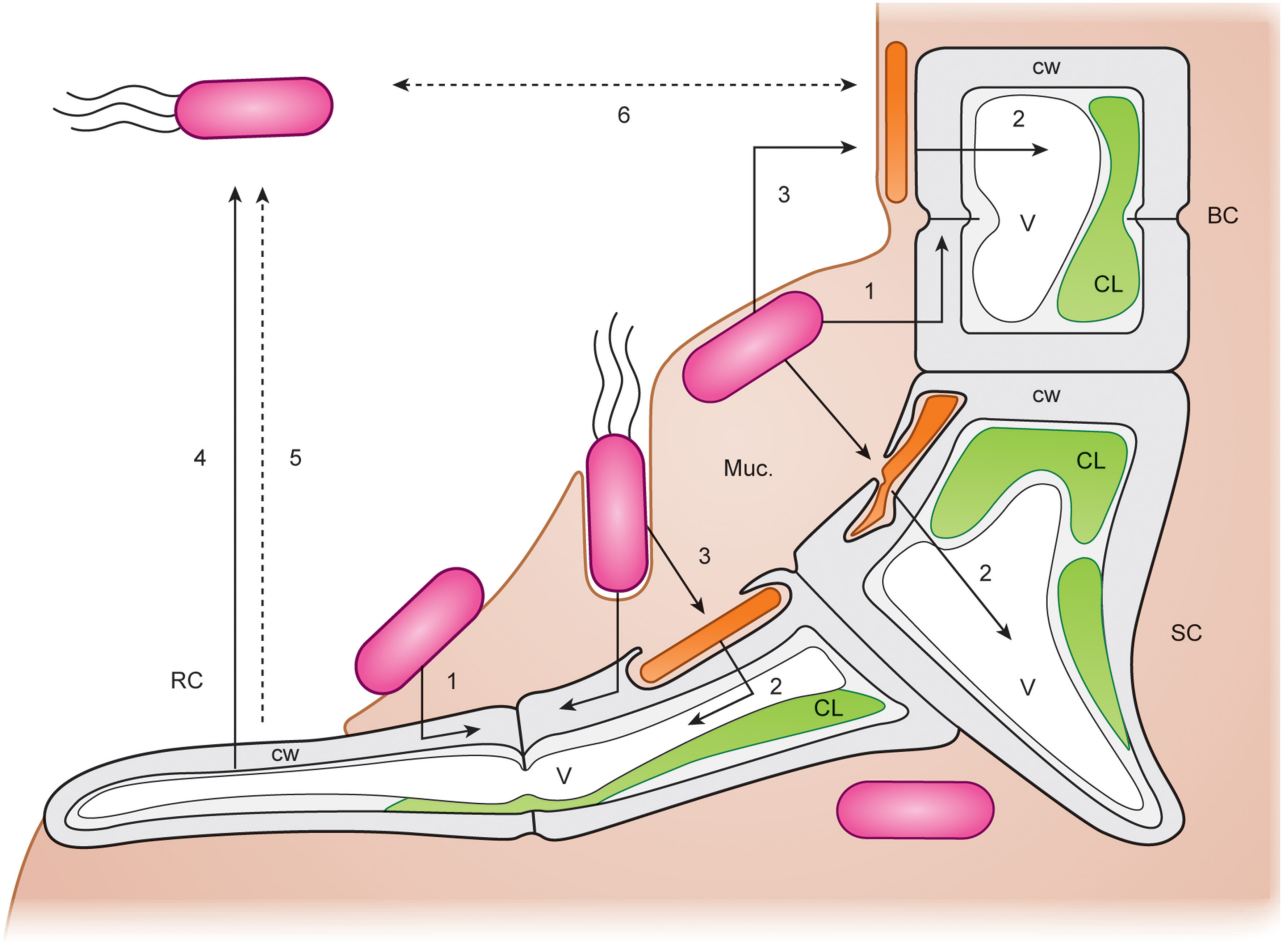
**Potential cross-kingdom interactions**. Detailed working model for the interactions of *Ulva mutabilis* with *Cytophaga* sp. (MS6) (orange rods) and *Roseobacter* sp. (MS2) (pink rods with or without flagellae). Arrows indicate possible chemical interactions. BC, blade cell; SC, stem cell; RC, rhizoid cell of *U. mutabilis* (wildtype) germling. CW, algal cell wall; V, vacuole; C, chloroplast; Muc, mucilage in which the bacteria are embedded. (1) *Roseobacter* stimulates *Ulva* cell divisions and (2) *Cytophaga* induces *Ulva* cell growth stimulating vacuole extension by released morphogenetic regulators and promotes rhizoid and correct cell wall formation. (3) *Roseobacter* may promote *Cytophaga* viability. (4) Rhizoid cells of *U. mutabilis* produce a factor attracting *Roseobacter* by chemotaxis. (5) Antibiotic substances produced by the alga control growth of inappropriate bacteria and other epiphytes (Hornsey and Hide, 1985). (6) Quorum-sensing system of *Roseobacter* and *Cytophaga* mutually regulates the appropriate bacterial cell densities (adapted, colored and used with permission from Spoerner et al., 2012; Copyright© 2012, Wiley).

Spoerner et al. (2012) introduced a working model (Figure 8): The rod-shaped bacterium *Cytophaga* sp. may be recruited by incidental direct contact with the algal cell surface, where they may move by gliding on a mucilage layer. *Cytophaga* species are known to recognize and adhere specifically to surfaces. *Cytophaga* might then metabolize epiphytically algal polysaccharides or even invaginate into the cell wall. Indeed, bacteria were recently identified by TEM in *Ulva flexuosa* subsp. *pilifera* (Messyasz et al., 2013). Rhizoid cells have to attach to the substratum by an adhesive polyglycoprotein (Callow and Callow, 2006) for the formation and maintenance of this cross-kingdom assembly. Subsequently, germinating *Ulva* excrete a diffusible substance, such as a specific nutrient or regulatory factor. Motile *Roseobacter* cells are capable of responding to the signals and move towards the germling and settle where the holdfast will be developed. They successively assemble and deposit a layer of self-produced mucilage. The cross-talk between axenic, very young germlings, and bacteria results in the formation of a biofilm in its most simplistic form under these controlled conditions for *U. mutabilis* (Figure 7). Extracellular polymeric substances can then facilitate the primary settlement of zoospores that play a crucial role in the recruitment of *U. fasciata* (Singh et al., 2013). This mucilage may form the matrix of an organized biofilm (Figures 7, 8). Multiple chemical interactions are proposed necessary for the establishment of a biofilm and, subsequently, induction of algal morphogenesis (Spoerner et al., 2012). This certainly simplified, but standardizable, system can be easily adjusted to further research lines (Egan et al., 2014; Singh and Reddy, 2014) related to grazing (chemical defense), effects of light/temperature (environmental conditions), and physical stresses on the community.

### Stochastic selection of bacteria by *Ulva*

As *Ulva* is symbiotic and bacteria-inducing morphogenesis seems to be, in part, a species-dependent process, the algae might structure their complex community actively. In this context, Burke et al. (2011a) analyzed the relationship between the bacterial community structure and their functions, using an elaborate metagenomic approach. Interestingly, they found a very high similarity in “*functional composition*” despite the low phylogenetic similarity (less than 15%) between the bacterial communities extracted from various *U. australis* samples. The authors concluded that a core suite of functions of genes has to be present *“independent of the taxonomic or phylogenetic composition”* of the species (Burke et al., 2011a). A core microbial community does not exist in *Ulva* species, as all microbiota vary over the season and from tidal pools in very close proximity (exemplified for *Ulva australis*). Therefore, the assemblage of epibacteria might be determined rather by a “lottery” than controlled by mechanistic (i.e., mutualistic) interactions (Burke et al., 2011a,b). In the context of the morphogenetic compounds, Burke's hypothesis (2011) was supported by the activity of the *Roseobacter*-factor, which can be provided by various bacteria strains, including the γ-Proteobacterium *Halomonas* sp. (Marshall et al., 2006; Spoerner et al., 2012). Even more interesting, boiling extracts of certain bacteria, such as *E. coli*, provide surprisingly the same activity, implying that the metabolite is produced, but not released, as the morphogens could not be determined in the medium. Solely metagenomic-based approaches might hence also overestimate the functions provided by the microbiome, as the complete signal transduction cascade has to be reconstituted starting from the biosynthesis of the compound till their release in order to prove the ecophysiological meaning for *Ulva*.

Taxon independent species but with identical functional traits occupy the same niche of *Ulva*'s surface, facilitated through stochastic chances. Different species within these guilds should share the equal ecophysiological functions and bear, consequently, a core suite of functional genes (i.e., biosynthetic pathways) (Burke et al., 2011a,b), which should be consistently present in the community of *Ulva* in its particular habitat at a given time, for example, during zoospore settlement and, subsequently, germination. This might question how we interpret the recruitment of the bacteria by macroalgae. Indeed, various studies have indicated that the bacterial surface community is different from the open seawater community (Lam and Harder, 2007; Lachnit et al., 2009; Sneed and Pohnert, 2011a), which implies a selective recruitment controlled by a host-specific enforced selection mechanism typical for mutualistic host-microbe systems (Lachnit et al., 2010; Sneed and Pohnert, 2011b; Friedrich, 2012). Bacteria-induced morphogenesis and QS signaling between zoospores and bacteria point out the necessity of very specific signal molecules/infochemicals, which are species-specific and selectively perceived by the algae. In view of a cross-kingdom cross-talk, bacteria are not only emitters of specific compounds perceived by the alga, they also receive and process potential (chemotactic) signal molecules, which might be released by the alga [e.g., dimethylsulfoniopropionate (DMSP): Miller et al., 2004; Kerrison et al., 2012], but further evidences are necessary.

In summary, regarding the *Roseobacter*-factor, *U. mutabilis* can easily gamble for the bacteria, but the *Cytophaga-*factor could not be provided by another bacterium and seems to be host-specific (up to now). Members of the α-Proteobacteria and the Bacteroidetes are part of the eco physiologically important stable subpopulations of the epiphytic microbial community demonstrated by denaturing gradient gel electrophoresis studies (Tujula et al., 2010; Burke et al., 2011a) and by bioassay-driven experiments (Marshall et al., 2006; Spoerner et al., 2012).

## Concluding remarks

Elaborative “omics” approaches (e.g., metagenomics, transcriptomics, and metabolomics) and classic tedious laboratory experiments have to be combined in order to understand the specific mechanisms between macroalgae and their associated bacteria. Hereby, it is important to note that standardized cultivation conditions are a necessary prerequisite for reliable experiments. This includes the time-consuming cultivation of macroalgae, starting with the preparation of axenic germ cells as a feed stock. To make life easier, the life cycle of *Ulva* should be fully controlled by induction/inhibition of sporulation and well-defined symbiotic bacteria have to be administered. Artificial seawater medium has to be checked to see whether the additives always meet the requirements of the bacteria essential for algal growth. Starting from the inspiring experiments by Provasoli (1958a,b) and the mass isolation of morphogenesis-inducing bacteria in Ulvales by Matsuo et al. (2003) and Nakanishi et al. (1996) (Table 1), the stable tripartite community of *U. mutabilis* (Figure 5) is suggested to unravel the cross-kingdom cross-talk of alga and bacteria. Interestingly, the microbiome could be reduced to only two species, which release the essential morphogenetic compounds. The bacteria of this minimal working mutualistic community are fortunately cultivable. *Ulva* can even develop into a foliose thallus without any bacteria, but with partially purified morphogens in its presence, which might be interesting for distinguishing between algal and microbial waterborne compounds. If those compounds are not available, as they are not yet characterized and can only be obtained in very small amounts from the growth medium, two-chamber systems can provide axenic algal cultures with a normal morphotype. These studies will also help to differentiate between the infochemical-mediated vs. cell-to-cell-based communication.

In the midterm, all these results gathered in the last few decades will be of further interest, if genetic analyses are carried out along with genome sequencing of *Ulva*. Both are underway and, therefore, newly generated morphotypes of *Ulva*, for example, by insertional mutagenesis, will shed light on the underlying mechanism of the bacteria-ruled morphogenesis in Ulvales (Wichard et al., 2015), but also on the unicellular–multicellular transitions in seaweeds (Coates et al., 2014). Advances in analytical chemistry instrumentation along with simplified data processing makes metabolomic approaches into a valuable tool in chemical ecology. Although great progress has been made in the last two decades since Bradley (1991) critical reviewed the role of hormones in controlling algal growth and development, researchers still have a long way to go to address the tedious questions of cross-kingdom interactions regarding the biosynthesis, bacterial release, algal perception, and signal transduction of morphogenetic compounds. Classic molecular/biochemical approaches (e.g., algal genetics) are only now becoming available and have to be applied in an orchestrated interdisciplinary consortium of laboratories, each providing their different key expertise, in order to unravel the underlying mechanisms of macroalgal growth in a symbiosis and to build up theories on host-microbe interactions in green seaweeds.

### Conflict of interest statement

The author declares that the research was conducted in the absence of any commercial or financial relationships that could be construed as a potential conflict of interest.

## Footnotes

^1^UTEX. (2014). http://web.biosci.utexas.edu/utex/mediaDetail.aspx?mediaID=38 [Online]. University Texas (Accessed April 11, 2014).
